# Association between chronic obstructive pulmonary disease and osteoporosis: Mendelian randomization combined with bibliometric analysis

**DOI:** 10.1186/s41065-025-00373-z

**Published:** 2025-02-01

**Authors:** Fangjun Yang, Huaming Wang, Miaomiao Liu, Shengtai Pei, Xiaoming Qiu

**Affiliations:** 1 Department of Orthopedic, Gansu Provincial Hospital of Traditional Chinese Medicine (The First Affiliated Hospital of Gansu University of Traditional Chinese Medicine), Lanzhou, 730050 China; 2 Gansu University of Traditional Chinese Medicine, Lanzhou, 730050 China

**Keywords:** Mendelian randomization, Chronic obstructive pulmonary disease, Osteoporosis, Bibliometric analysis, CiteSpace, Relationship

## Abstract

**Background:**

Previous observational studies have reported an association between chronic obstructive pulmonary disease (COPD) and osteoporosis (OP). The aim of this study is to investigate the causal relationship between COPD and OP by two-sample Mendelian randomization (MR) analysis. The current status of cross-sectional research between COPD and OP in the past decade was explored through bibliometrics.

**Methods:**

Single nucleotide polymorphisms (SNPs) that have been found to be strongly associated with COPD were used as instrumental variables (IVs) in MR Analysis. The primary outcome of the study was BMD measurement at five specific anatomical sites, namely the whole body, femoral neck, lumbar spine, forearm, and heel. These BMD measurements were derived primarily from a genome-wide association study (GWAS) and summary statistics from the International Genetic Factors Consortium for Osteoporosis (GEFOS). The main analysis method was inverse variance weighting (IVW). Multiple sensitivity analyses were performed to assess the robustness and reliability of the current MR Results. Further confirmatory MR Analysis between COPD and OP was applied. In bibliometrics. Publications were extracted from the Web of Science core collection publications related to osteoporosis and sarcopenia published between January 2014 and October 2024; Bibliometrics and visualization were performed by Microsoft Office Excel, Citespace, and R (Bibliometrix).

**Results:**

The MR Findings suggest that there is no causal relationship between COPD and BMD at five specific anatomical sites. The results of the primary IVW MR Analysis were generally supported by our sensitivity MR Analysis. We performed MR Analysis for the validation of COPD and OP (IVW OR: 1.019; 95%CI: 0.898–1.564; *p* = 0.768) also did not support a causal relationship between COPD and BMD. A total of 471 articles written by 1119 organizations from 42 countries/regions by 3331 authors and published in 238 journals were identified in the bibliometric analysis. China is the leading country in terms of the number of publications. China Medical University contributed the most publications. The International Journal of Chronic Obstructive Pulmonary Disease has the highest publication in this field.

**Conclusions:**

In conclusion, This MR Study found no causal relationship between COPD and OP, suggesting that the observed associations may be due to common genetic effects or environmental confounders. The global research trends in this field in the past decade were summarized through bibliometric analysis, and care became the focus of future research on the relationship between copd and OP.

**Supplementary Information:**

The online version contains supplementary material available at 10.1186/s41065-025-00373-z.

## Introduction

Osteoporosis (OP) is a systemic skeletal disorder that is characterized by a reduction in bone mass and the deterioration of bone tissue's microstructure. This condition leads to a decrease in bone strength and an increased susceptibility to fragility fractures [1, 2]. The diagnosis of osteoporosis is dependent on the evaluation of bone mineral density (BMD), which is commonly determined through the utilization of dual-energy X-ray absorptiometry. According to the Health Organization, OP is characterized by a BMD value that is lower than 2.5 standard deviations from the average BMD of young individuals who share the same race and gender [3]. Currently, the prevalence of OP is reported to be 18.3%, with notable variations observed among different ethnic groups and geographical locations [3, 4]. As the global population continues to age, there has been a steady increase in the prevalence of OP [5]. Numerous risk factors are associated with the development and progression of OP. These risk factors can be categorized into non-modifiable factors, such as age, ethnicity, gender, and family history, and modifiable factors, including smoking habits, alcohol abuse, weight loss, inadequate nutrient absorption, reduced physical activity, and certain chronic diseases [6, 7]. Therefore, it is crucial to examine the relationships linked to BMD.

Chronic obstructive pulmonary disease (COPD) is a prevalent and incurable, yet manageable, progressive condition [8]. It is distinguished by the existence of enduring airflow restriction, primarily resulting from structural alterations in the trachea and chronic inflammation. This condition ultimately results in a gradual deterioration of pulmonary function, diminished exercise capacity, and the emergence and advancement of extrapulmonary complications [8]. Airflow limitation can be commonly detected using spirometry and categorized based on the criteria established by the Global Initiative for Chronic Obstructive Lung Disease (GOLD). According to these criteria, airflow limitation is identified when the ratio of forced expiratory volume in one second (FEV1) to forced vital capacity (FVC) is less than 80% of the predicted value, and when FEV1 is less than 80% of the predicted value [9, 10]. Currently, COPD is acknowledged as the third most prevalent cause of mortality worldwide, ranking behind ischemic heart disease and stroke [8]. Recent research findings indicate that individuals who have been diagnosed with COPD have a significantly elevated risk of developing OP when compared to the general population. This increased risk can range from two to five times higher [11, 12]. Multiple studies have demonstrated a correlation between these two conditions as a result of common risk factors and the presence of inflammatory manifestations, which are intricately linked through shared pathophysiology. However, it remains unclear whether there is a definitive answer to this question [13–15].

Mendelian randomization (MR) is an epidemiological method to infer causal relationships. This study mainly uses genetic variants, especially single nucleotide polymorphisms (SNPs) as instrumental variables (IVs) to assess the potential causal effect of exposure on outcomes [16–18]. Bibliometrics refers to the study that uses statistics to describe publication trends and highlight relationships between published works [19]. It is helpful for researchers to understand the progress trend and key areas of related research, determine research direction, and explore new research focus [20].

In this study, we aimed to examine the causal relationship between COPD and OP by identifying SNPs associated with COPD and using them as IVs. The association between COPD as exposure and OP as outcome was assessed using confirmatory MR Analysis. Subsequently, the literature in the intersection field of COPD and OP from 2013 to 2024 was analyzed, and the bibliometric method was used to understand the current trends and topics, so as to provide a comprehensive overview for researchers to explore and further research in this field.

## Methods

### Mendelian randomization

#### Research design

**Fig. 1:**
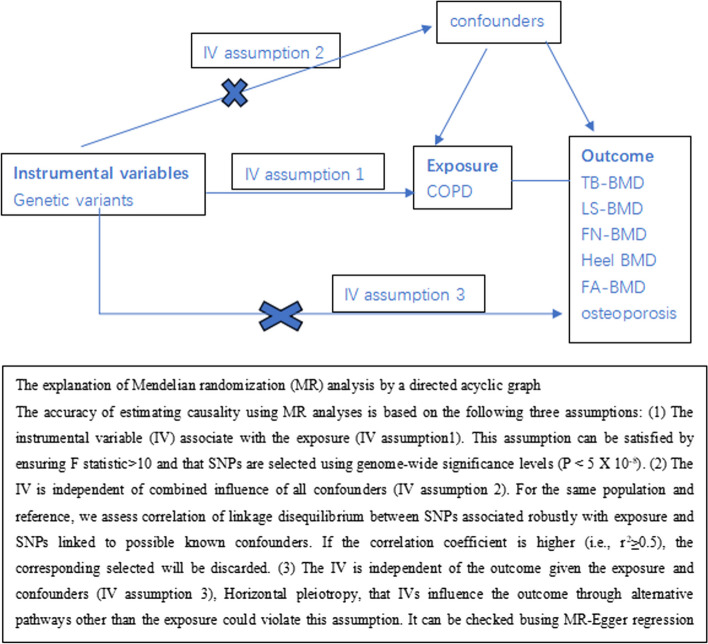
Directed acyclic graphs for the classical Mendelian randomixation designs

In this study, a two-sample MR method was employed (Fig. 1),

### Data sources

#### Exposure (Chronic obstructive pulmonary disease)

We employed a total of 82 SNPs that were identified by Sakornsakolpat as being associated with COPD. The research encompassed a comprehensive sample size of 35,735 cases and 222,076 control [21]. The data were derived from a comprehensive sample of 257,811 individuals, encompassing 25 meta-analyses of GWAS. In addition, prior studies have performed MR analysis on this dataset [22] (supplementary Table 1).

#### Outcome (bone mineral density, osteoporosis)

The outcome data used included total body bone mineral density (TB-BMD), lumbar spine bone mineral density (LS-BMD), femoral neck bone mineral density (FN-BMD), heel bone mineral density (Heel BMD), and forearm bone mineral density (FA-BMD). The validation outcome data indicated osteoporosis. All the above data were collected from the statistical data of GEFOS (http://www.gefos.org/) and IEU Open GWAS. Ethical approval and informed consent were obtained. The writing process followed the requirements of STROBE-MR, the reporting standard for MR research. Details of the data are shown in Table 1.

**Table 1:** BMD GWAS data summary information

GWAS ID	Year	Trait	Consortium/Author	Sample size	Number of SNPs
ebi-a-GCST00 5348	2018	Total body BMD	GWAS meta-analysis	56 284	16 162 733
ieu-a-982	2015	Lumba spine BMD	GEFOS	28 498	10 582 867
ieu-a-980	2015	Femoral neck BMD	GEFOS	32 735	10 586 900
ieu-a-977	2015	Forearm BMD	GEFOS	8 143	9 955 366
ebi-a-GCST00 6979	2019	Heel BMD	GEFOS	426 824	13 705 641
finn-b-M13_OSTEOPOROSIS	2021	Osteoporosis	Finn Gen	212 778	16 380 452

### Selection of instrumental variables (IVs)

In this study, SNPs meeting the following criteria were selected as IV [16–18]. (1) SNPS highly associated with COPD (*p* < 5 × 10^–8^). (2) To avoid linkage disequilibrium (LD) caused by SNPs associated with COPD, LD must meet *r*^2^ < 0.001 and kb = 10,000. (3) To ensure a strong relationship between IV and COPD, we chose an F statistic > 10 to avoid bias caused by a weak IV. F statistic = (β/SE)^2^ [23]. And to test the second MR hypothesis that genetic variants are independent of potential confounders, we conducted a search in the PhenoScanner database [24, 25]. To exclude IV that are associated with known confounders such as sex, lifestyle, advanced age, steroid therapy, low body mass index (BMI), hormonal imbalance, and certain chronic diseases.

### Mendelian randomization analysis

In this two-sample MR Design, we employed several methods to investigate the causal association between genetic variants related to COPD and BMD. These methods included the inverse variance weighting (IVW) method, weighted median (WM) method, simple median (SM) method, weighted median estimator (WME), and MR-Egger regression [26–28]. Among the various methods employed, the inverse variance weighting (IVW) method served as the main method to calculate odds ratios (OR). The outcome estimate was represented by the slope of the weighted regression of the outcome effect on the exposure effect, with an intercept restricted to zero. Furthermore, the robustness of the primary outcome was assessed using four additional methods.

### Sensitivity analysis

We adopted a distinct methodology to mitigate potential confounding factors, thereby ensuring that the independent IV remained unaffected by outcomes other than the exposure. Firstly, the researchers utilized the Pleiotropy Residual Sum and Outlier (MR-PRESSO) method to examine and adjust for any outliers related to horizontal pleiotropy. Additionally, any outliers detected in the instrumental variable (IV) were eliminated [26]. We employed MR-Egger regression as a statistical method to elucidate the presence of horizontal pleiotropy. When the *P* value of the intercept exceeds 0.05, it indicates the absence of horizontal pleiotropy [29]. Horizontal pleiotropy was assessed through the utilization of a funnel plot. If the funnel plot exhibits a symmetrical shape, it typically indicates the absence of apparent pleiotropy.

The heterogeneity of individual estimates of genetic variation was evaluated through the application of Cochran's Q test. If the *p*-value of Cochran's Q test is > 0.05, it indicates that there is no statistically significant heterogeneity among SNPs. In this case, both the random effect model and fixed effect model can be utilized, as the results obtained from these two methods are comparable [30]. The *p*-value of Cochran's Q test was found to be less than 0.05, suggesting the presence of heterogeneity among SNPs. Therefore, it is necessary to conduct MR analysis using the random effects model. Therefore, the random effects model with inverse variance weighting method was employed for MR Analysis in this study [31, 32]. The leave-one-out sensitivity test is employed to assess the impact of removing specific SNPs on the overall results, thereby determining if there are any significant changes. If individual SNPs are systematically removed, the results consistently demonstrate a relatively stable pattern, suggesting that the overall range of error in the data remains small even after the removal of any single SNP. Specifically, a stepwise approach was employed to remove each SNP one by one. Subsequently, the meta-effects of the remaining SNPs were computed. The individual influence of each SNP was assessed separately using IVW analysis and visually represented through a forest plot.

### Analysis software

All statistical analyses were conducted using R Studio (version 4.3.1), two-sample MR (version 0.5.7), and MR-PRESSO (version 1.0) software packages [18]. Results are reported in the form of odds ratios (ORs) accompanied by 95% confidence intervals (95% CI). Statistical significance was determined at a *p*-value of less than 0.05.

## Bibliometric analysis

### Data source and retrieval strategy

The data we collected came from the Science Citation Index Expanded (SCI-EXPANDED) and Social Science Citation Index (SSCI) of the core database of Web of Science. The database is currently the most commonly used database for scientometrics analysis, providing a variety of authoritative and high-impact academic journals, and is an important source of global authoritative academic information [33].

We searched and downloaded all literature identified from WoSCC's SCI-Expanded from January 1, 2014 to 2024–09-30. The specific retrieval methods were TS = (osteoporosis OR osteopenia OR osteoporotic OR bone loss OR low bone mass OR low bone density) AND TS = (chronic obstructive pulmonary disease OR COPD OR chronic airflow obstruction OR chronic obstructive lung disease), The following documents types were excluded abstracts, editorial materials, proceedings papers, corrections, letters, book chapters, published online.and news, meeting.

### Data analysis and visualization

After the screening and extraction steps, the final number of included articles was 471. CiteSpace [34], Excel and Bibliometrix [35] were used for data analysis and visualization.Bibliometric data include year of publication, title, author name, nationality, unit, abstract, keyword, journal name.

## Result

### Genetic tools for COPD

SNPs (*P* < 5 × 10^–8^) and linkage disequilibrium parameters (*r*^2^ < 0.001, kb = 10,000) were considered in the analysis. The F value of each SNP was calculated to be greater than 10, effectively mitigating the potential influence of weak IV on MR Analysis [36]. The analysis was conducted on the remaining 59 SNPs (Supplementary Table 2).

### Causal relationship between COPD and BMD

**Fig. 2:**
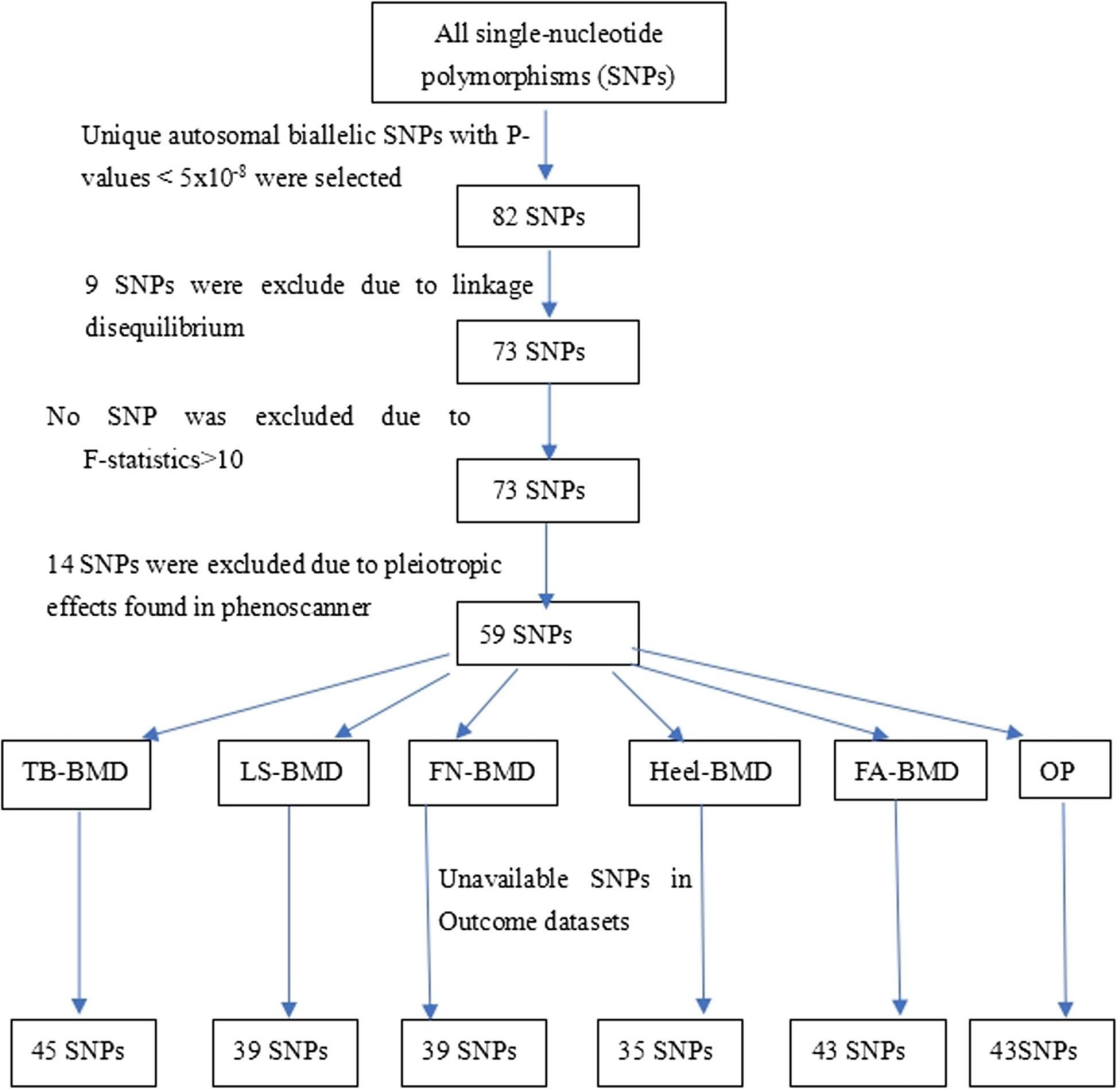
The flowchart of instrumental variables selesction. BMD, bone mineral density; TB, Total body; LS, Lumbar spine; FN, Femoral neck; FA; OP, osteoprosis

BMD results, including TB-BMD, LS-BMD, FN-BMD, Heel-BMD, and FA-BMD, were obtained from GWAS data. To mitigate potential confounding factors, any missing and proxy SNPs were excluded from the analysis. Additionally, the exposure and outcome datasets were merged, and any palindromic sequence SNPs that were detected were subsequently eliminated. The PhenoScanner database was employed to eliminate SNPs that may be influenced by confounding factors associated with osteoporosis. Additionally, MR-PRESSO was utilized to identify and exclude any potential outliers. A total of 45 phenotypes were identified as having a potential causal relationship with whole body BMD, while 39 phenotypes were found to have a potential causal relationship with lumbar spine BMD. Additionally, 39 phenotypes were identified as having a potential causal relationship with femoral neck BMD, and 43 phenotypes were found to have a potential causal relationship with forearm BMD. There are 35 phenotypes that exhibit potential causal relationships with heel BMD (Supplementary Table 3–8) (Fig. 2).

Our MR Study did not identify any conclusive evidence of a causal relationship between COPD and BMD at the five sites (Table 2). TB-BMD (OR = 0.988, 95%CI: 0.958–1.019, *P* = 0.464), LS-BMD(OR = 0.997, 95%CI: 0.948–1.049, *P* = 0.928), FN-BMD(OR = 1.009, 95%CI: 0.958–1.049, *P* = 0.928), TB-BMD (OR = 0.988, 95%CI: 0.958–1.019, *P* = 0.464) 0.973–1.046, *P* = 0.603), Heel BMD(OR = 1.01, 95%CI: 0.996–1.023, *P* = 0.131), FA-BMD(OR = 0.959, 95%CI: 0.891–1.032, *P* = 0.264).

**Table 2:** Two-sample MR results of COPD on Osteoprosis

		BMD(outcome)				
		TB-BMD	LS-BMD	FN-BMD	Heel BMD	FA-BMD
No.SNPs used		45	39	39	35	43
IVW	OR	0.988	0.997	1.009	1.01	0.959
	95%CI	0.958–1.019	0.948–1.049	0.973–1.046	0.996–1.023	0.891–1.032
	*P* value	0.464	0.928	0.603	0.131	0.264
Weighted median	OR	0.997	0.997	1.035	1.011	0.963
	95%CI	0.957–1.038	0.937–1.062	0.981–1.091	0.995–1.027	0.869–1.066
	*P* value	0.893	0.946	0.203	0.148	0.471
Weighted mode	OR	0.979	0.997	1.052	1.015	1.057
	95%CI	0.906–1.059	0.88–1.13	0.946–1.171	0.984–1.047	0.85–1.315
	*P* value	0.611	0.971	0.352	0.346	0.616
MR Egger	OR	0.97	0.958	0.981	1.029	0.974
	95%CI	0.836–1.126	0.752–1.219	0.824–1.167	0.965–1.098	0.684–1.387
	*P* value	0.698	0.729	0.834	0.382	0.887
Simple mode	OR	0.988	1.051	1.058	1.015	1.054
	95%CI	0.91–1.073	0.919–1.202	0.952–1.175	0.98–1.051	0.839–1.324
	*P* value	0.784	0.466	0.297	0.393	0.652

We further confirmed our findings using weighted median, weighted mode, and simple mode MR (Table 2). MR-Egger causal estimation yielded similar results, with a wider CI for MR-Egger than for IVW, consistent with the lower statistical power of this test. The *P* values of the intercept in the corresponding MR-Egger regression were all higher than 0.05, indicating that there was no evidence of pleiotropy (Table 2). The MR-PRESSO test identified a total of 10 abnormal SNPs that were associated with the outcome of Heel BMD. These SNPs include rs11655567, rs117261012, rs13140176, rs1529672, rs2442776, rs34727469, rs62065216, rs11655567, rs117261012, rs13140176, rs1529672, rs2442776, rs34727469, rs62065216, rs798565, rs803923, and rs9399401. After excluding outliers, repeated MR Analysis again showed no evidence of causal relationship between genetically determined COPD and Heel BMD (Table 2).

In addition, a leave-one-out analysis was conducted on the BMD results at each of the five sites. None of the SNPs exhibited a statistically significant influence on the overall causal estimates (Supplementary Figs. 1–5). To enhance the comprehensiveness of the aforementioned findings, a thorough investigation of horizontal pleiotropy and heterogeneity was carried out by employing funnel plots and scatter plots. The funnel plots exhibited a symmetrical distribution of causal effects, without any apparent bias observed (Supplementary Figs. 6–10). Scatter plots provided additional evidence to support the lack of a causal association between COPD and BMD at the five specified anatomical sites (Supplementary Figs. 11–15).

### Causal relationship between COPD and OP

To enhance the robustness of the results, a confirmatory analysis was performed on a two-sample MR Between COPD and OP. We have successfully identified 43 SNPs that exhibit a potential causal association with OP (Fig. 2). The results from the MR analysis did not indicate any causal relationship between the two variables (Table 3). (IVW OR: 1.019; 95%CI: 0.898–1.564; *p* = 0.768). The stability of the results was demonstrated by the remaining four methods. We generated a scatter plot to examine the impact of each SNP individually exposed on the outcome SNP. In the plot, the colored lines corresponded to different statistical methods. Notably, all five MR methods yielded consistent results. Our analysis revealed that there was no observed effect of increasing COPD on OP (Fig. 3①).

**Table 3:** Two-sample MR results of COPD on Osteoprosis

No. SNPs used	44	
IVW	OR per SD (95%CI)	1.019 (0.898–1.564)
	*P* value	0.768
	Q_*p*-value	0.247
Weighted Median	OR per SD (95%CI)	1.083 (0.915–1.281)
	*P* value	0.35
Weighted mode	OR per SD (95%CI)	1.174 (0.877–1.571)
	*P* value	0.285
MR-Egger	OR per SD (95%CI)	1.285 (0.714–2.311)
	*P* value	0.406
Simple mode	OR per SD (95%CI)	1.105 (0.793–1.54)
	*P* value	0.556

**Fig. 3:**
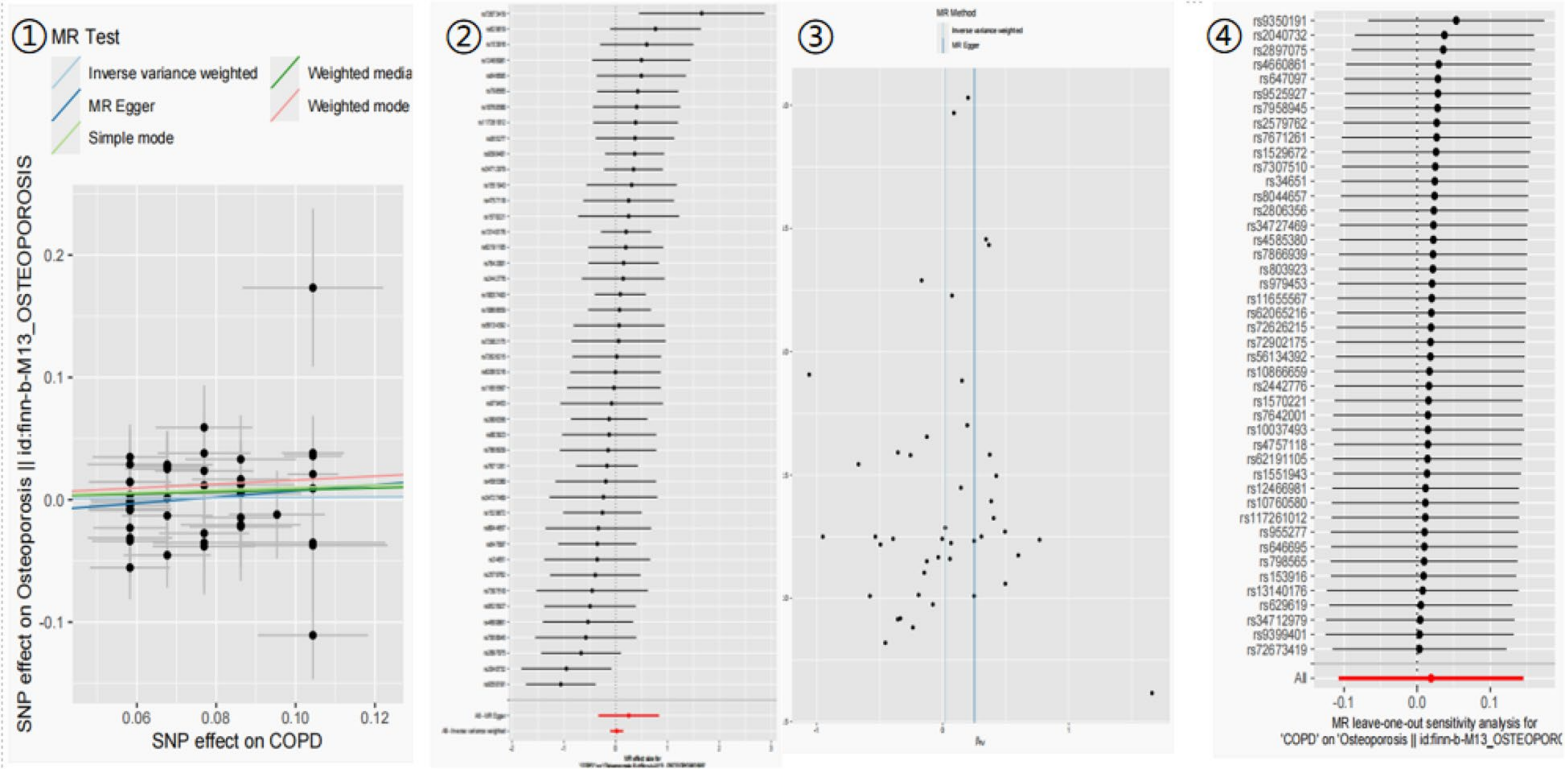
Mendelian randomization plot. ①. Causal association diagram between COPD and OP ②. Forest plot of MR of COPD and OP ③. Funnel plot of MR for COPD and OP ④. leave-one-out plot of MR for COPD and OP

We employed a single SNP analysis approach to assess the impact of each SNP that is linked to COPD on OP, (Fig. 3.②). SNP rs72673419 may be considered an outlier due to its significant impact on both COPD and OP. Despite the omission of this SNP from the analysis, the findings remained consistent. (Supplementary Fig. 16). The distribution of causal effects depicted in the funnel plot exhibited a predominantly symmetric pattern, indicating the absence of significant bias (Fig. 3③). Additionally, the leave-one-out test analysis demonstrated that none of the (SNPs had a noticeable effect on the overall causal estimate (Fig. 3④).

## Visual analysis of articles

According to the search strategy, 471 articles on the relationship between COPD and OP were finally obtained from 2014 to 2024. There were 373 original studies and 87 reviews. Since 2013, the annual number of articles published between COPD and OP was maintained between 30 and 60. As shown in the Fig. 4①, the number of publications between copd and osteoporosis remained stable over the last decade.

**Fig. 4:**
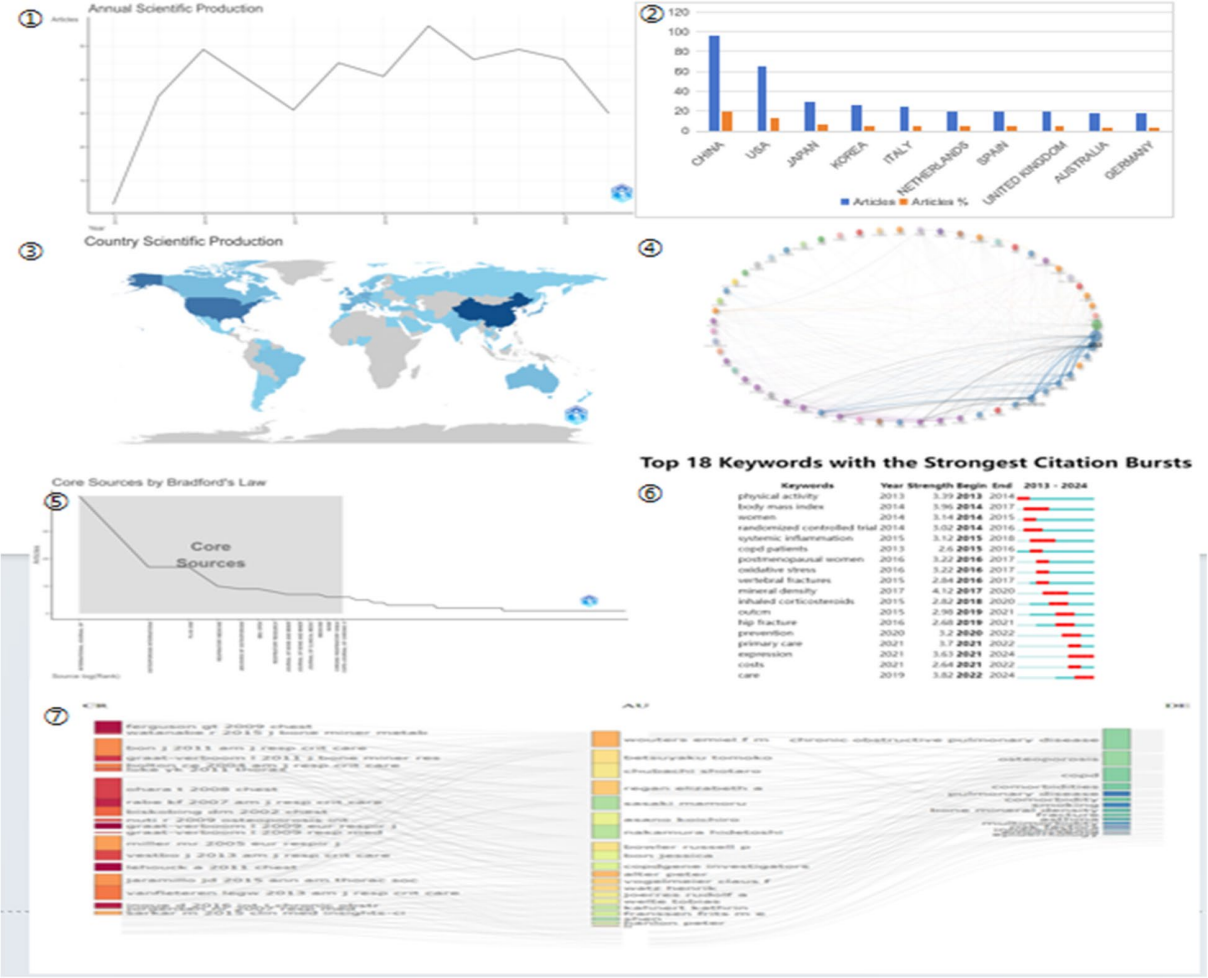
Bibliometric analysis of COPD and OP

### Visual analysis of countries and institutions

One thousand one hundred ninety academic institutions from 42 countries have published papers on the relationship between COPD and OP in the field of research.As can be seen from the Fig. 4②, China has published the most papers in this field, with 96 publications, accounting for 20.4% of the total, followed by the United States (65 publications, 13.8%), and Japan (30 publications, 6.4%). Other countries have fewer than 30 publications.

Figure 4③ shows the global distribution of all COPD and OP publications from 2014 to 2024, with more publications represented in dark blue, and we can see that more publications are concentrated in North America, Western Europe, and East Asia.The institutions that published the most papers were China Medical University (35,7.4%), followed by the affiliated hospitals of China Medical University. Obviously, the top two institutions were both in China. From Table 2, we can see that half of the top ten institutions in the field of global output are in China, and the rest are in three developed countries, the United States, the United Kingdom and Japan. Figure 4④ shows the distribution of cooperation between different countries, and the width of the connection between two countries indicates the strength of cooperation. It can be seen from the Fig. 4④ that the countries with the most publications have the closest cooperation, such as the United States, China, and the United Kingdom.

### Visual analysis of authors, journals and keywords

A total of 3331 authors participated in the study of the association between COPD and OP. Among them Wouters emiel (*N* = 7), Regan Elizabeth (*N* = 6) and Vogelmeier Claus (*N* = 6) published the most papers.

The above articles have been published in 238 journals. According to Bradford's Law, 14 journals are identified as core journals, of which the most published is the International Journal of Chronic Obstructive Pulmonary Disease, with 18 journals publishing at least five articles (Fig. 4⑤). 1114 author keywords were extracted from 471 articles. Chronic obstructive pulmonary disease, copd and osteoporosis had the highest incidence at 101,86 and 125, respectively. In addition, we applied CiteSpace's burst detection algorithm to display the top 18 keywords with the highest burst intensity, as shown in Fig. 4⑥. Finally, we constructed a three-field graph to look at the relationship between journals, authors, and keywords (Fig. 4⑦), and the results are consistent with the above analysis.

## Discussion

With the increase of global aging, the incidence of COPD and OP co-morbidity is increasing. Along with the increase in the number of studies, it is necessary for researchers to have a comprehensive understanding of the changes in global research trends in the field. We combined MR Methods and bibliometric analysis to explore the potential association between COPD and BMD, and visualized the research hotspots between the two in recent years.

There was no direct causal association between COPD and BMD in the MR Study. We performed several sensitivity analyses to distinguish true negative results from those lacking validity, thereby ensuring that the study met the three MR Assumptions. Given the use of the methods described above to demonstrate the consistency of the results, we have high confidence in the accuracy of the results obtained from the MR Analysis. To determine the robustness of our findings, we used a large dataset of osteoporosis information from the FinnGen Consortium to confirm our results, thus increasing the credibility of the study.

However, some previous studies have demonstrated a correlation between COPD and BMD, which is in stark contrast to our findings [15, 37–39]. The most compelling study in this field is the comprehensive systematic review and meta-analysis conducted by Ahmad Naoras Bitar et al. [15]. The study provides evidence for a positive association between COPD and decreased bone mineral density. However, it is important to note that most of the studies included in the meta-analysis were observational in nature. The observed association may be attributable to unmeasured confounders, such as common risk factors for COPD and bmd, including smoking, physical inactivity, falls, and lower educational attainment [6]. It is difficult to fully control the complex interactions among environmental, genetic, and lifestyle factors in observational studies, which may lead to confounding bias, and the accuracy of the above results is often questioned. The MR Method is to infer the causal relationship through the natural random allocation between genes and diseases, which is not affected by confounding factors and provides more reliable causal inference.Although there is no causal relationship between the two, for patients with COPD and osteoporosis, modifying the above controllable risk factors, such as avoiding smoking, moderately increasing physical activity, and avoiding falls, can effectively alleviate the symptoms of the disease and the complications caused by it, such as fractures, cor pulmonale, etc.

To further verify the credibility of our findings, we found that two randomized controlled clinical trials [40, 41] found a possible common association between COPD and BMD by osteoclast activation indicators such as matrix metalloproteinase activity. A recent bioinformatics study found that two similar mirnas (miR-23a-5p and miR-194-3p) were present in the peripheral blood of patients with COPD and OP [42]. These findings support the hypothesis that COPD and OP may share a common pathogenic molecular mechanism. However, these results do not suggest that COPD is more likely to cause bone mineral density loss or even osteoporosis. Taken as a whole, these data suggest a common genetic structure between BMD and COPD and may provide new possibilities for diagnosis and treatment of patients.

In addition, in the bibliometric study, we found that the number of publications worldwide remained stable in the past decade, indicating that the related research on COPD and OP is relatively mature. In terms of national contributions, China, the United States and Japan played an important role in the study of the relationship between COPD and OP, accounting for nearly 2/5 of the total number of publications, and the cooperation between these countries was the strongest. China published the largest number of articles, accounting for nearly one-fifth of the total. This shows that China is ahead of other countries in science and technology. This also shows that China attaches great importance to this field and increases investment. It has superior conditions in basic and clinical trials, professional technology and equipment, full-time researchers and sufficient scientific research funds, which makes it firmly occupy a leading position in the research field.

For the analysis of journals and keywords, we found the 18 journals with the most papers, and the International Journal of Chronic Obstructive Pulmonary Disease had the most papers. Through the key research, we found the 18 keywords with the highest burst intensity in the past decade, and care was the most concerned keyword in this research field. Currently, the focus on COPD and OP has shifted from the intersection of the two diseases to the point of convergence. Further research into the epidemiology, diagnosis, treatment and management strategies of both diseases is needed in the future.

The study has many advantages. First, we used MR Methods to explore the causal relationship between BMD and COPD for the first time, excluding potential confounding variables and reverse causality. Second, the consistency of results from different datasets obtained from the aggregated data of the GWAS, FinnGen and GEFOS consortiums guarantees the validity of our conclusions. Finally, based on bibliometrics, we analyze the evolution of research in this field in the past decade.

However, there are some limitations to our research. The database for the MR Study was drawn from people with European ancestry, so it is not obvious whether the findings apply to communities with non-European ancestry. Therefore, it is necessary to confirm these results in other populations. In addition, a significant flaw in the MR Study is the unobstructed pleiotropy, which may affect the interpretation of the genetic predictive relationship between COPD risk and BMD. The publications included in the bibliometrics method were all from SCI-E, and we excluded studies not included in SCI-E and studies not in English, which may lead to some omissions in the study.

## Conclusion

In conclusion, This MR Study found no causal relationship between COPD and OP, suggesting that the observed associations may be due to common genetic effects or environmental confounders. The global research trends in this field in the past decade were summarized through bibliometric analysis, and care became the focus of future research on the relationship between copd and OP.

## Supplementary Information

Supplementary Material 1.

Supplementary Material 2.

## Data Availability

No datasets were generated or analysed during the current study.

## References

[1] Deng Y, Wong MCS. Association between rheumatoid arthritis and osteoporosis in Japanese populations: a mendelian randomization study. Arthritis Rheumatol. 2023;75(8):1334–43. 37039764 10.1002/art.42502

[2] Yu XH, Yang YQ, Cao RR, et al. Rheumatoid arthritis and osteoporosis: shared genetic effect, pleiotropy and causality. Hum Mol Genet. 2021;30(21):1932–40. 34132789 10.1093/hmg/ddab158

[3] Pouresmaeili F, Kamalidehghan B, Kamarehei M, et al. A comprehensive overview on osteoporosis and its risk factors. Ther Clin Risk Manag. 2018;14:2029–49. 30464484 10.2147/TCRM.S138000 PMC6225907

[4] Salari N, Ghasemi H, Mohammadi L, et al. The global prevalence of osteoporosis in the world: a comprehensive systematic review and meta-analysis. J Orthop Surg Res. 2021;16(1):609. 34657598 10.1186/s13018-021-02772-0 PMC8522202

[5] Guo B, Wang C, Zhu Y, et al. Causal associations of brain structure with bone mineral density: a large-scale genetic correlation study. Bone Res. 2023;11(1):37. 37474577 10.1038/s41413-023-00270-z PMC10359275

[6] Deng YY, Liu YP, Ling CW, et al. Higher healthy lifestyle scores are associated with greater bone mineral density in middle-aged and elderly Chinese adults. Arch Osteoporos. 2020;15(1):129. 32804253 10.1007/s11657-020-00758-9

[7] Dougados M. Comorbidities in rheumatoid arthritis. Curr Opin Rheumatol. 2016;28(3):282–8. 27027814 10.1097/BOR.0000000000000267

[8] Lu K, Tan JS, Li TQ, et al. An inverse causal association between genetically predicted vitamin D and chronic obstructive pulmonary disease risk. Front Nutr. 2023;10:1111950. 37006939 10.3389/fnut.2023.1111950 PMC10050703

[9] Sin DD, Anthonisen NR, Soriano JB, et al. Mortality in COPD: role of comorbidities. Eur Respir J. 2006;28(6):1245–57. 17138679 10.1183/09031936.00133805

[10] Mannino DM, Thorn D, Swensen A, et al. Prevalence and outcomes of diabetes, hypertension and cardiovascular disease in COPD. Eur Respir J. 2008;32(4):962–9. 18579551 10.1183/09031936.00012408

[11] Inoue D, Watanabe R, Okazaki R. COPD and osteoporosis: links, risks, and treatment challenges. Int J Chron Obstruct Pulmon Dis. 2016;11:637–48. 27099481 10.2147/COPD.S79638 PMC4820217

[12] Akyea RK, Mckeever TM, Gibson J, et al. Predicting fracture risk in patients with chronic obstructive pulmonary disease: a UK-based population-based cohort study. BMJ Open. 2019;9(4):e024951. 30948576 10.1136/bmjopen-2018-024951 PMC6500346

[13] Cunningham TJ, Ford ES, Rolle IV, et al. Associations of self-reported cigarette smoking with chronic obstructive pulmonary disease and co-morbid chronic conditions in the United States. COPD. 2015;12(3):276–86. 25207639 10.3109/15412555.2014.949001 PMC4454614

[14] Shen L, Lv J, Li J, Zhou J, Wang X. Managing Osteoporosis in COPD. Endocrine, metabolic & immune disorders drug targets. 2024;24(8):896–901. 10.2174/1871530323666230913105752. 10.2174/1871530323666230913105752 37711118

[15] Bitar AN, Sulaiman SA, Ali IA, et al. Osteoporosis among patients with chronic obstructive pulmonary disease: systematic review and meta-analysis of prevalence, severity, and therapeutic outcomes. J Pharm Bioallied Sci. 2019;11(4):310–20. 31619912 10.4103/jpbs.JPBS_126_19 PMC6791086

[16] Davies NM, Holmes MV, Davey SG. Reading Mendelian randomisation studies: a guide, glossary, and checklist for clinicians. BMJ. 2018;362:k601. 30002074 10.1136/bmj.k601 PMC6041728

[17] Skrivankova VW, Richmond RC, Woolf BAR, et al. Strengthening the reporting of observational studies in epidemiology using mendelian randomisation (STROBE-MR): explanation and elaboration. BMJ. 2021;375:n2233. 34702754 10.1136/bmj.n2233 PMC8546498

[18] Hemani G, Zheng J, Elsworth B, et al. The MR-Base platform supports systematic causal inference across the human phenome. Elife. 2018;7:e34408. 29846171 10.7554/eLife.34408 PMC5976434

[19] Ninkov A, Frank JR, Maggio LA. Bibliometrics: methods for studying academic publishing. Perspect Med Educ. 2022;11(3):173–6. 34914027 10.1007/s40037-021-00695-4 PMC9240160

[20] Kokol P, BlažunVošner H, Završnik J. Application of bibliometrics in medicine: a historical bibliometrics analysis. Health Info Libr J. 2021;38(2):125–38. 31995273 10.1111/hir.12295

[21] Sakornsakolpat P, Prokopenko D, Lamontagne M, et al. Genetic landscape of chronic obstructive pulmonary disease identifies heterogeneous cell-type and phenotype associations. Nat genet. 2019;51(3):494–505. 30804561 10.1038/s41588-018-0342-2 PMC6546635

[22] Higbee D, Granell R, Walton E, et al. Examining the possible causal relationship between lung function, COPD and Alzheimer’s disease: a Mendelian randomisation study. BMJ Open Respir Res. 2021;8(1):e000759. 34233891 10.1136/bmjresp-2020-000759 PMC8264898

[23] Hemani G, Tilling K, Davey SG. Orienting the causal relationship between imprecisely measured traits using GWAS summary data. PLoS Genet. 2017;13(11):e1007081. 29149188 10.1371/journal.pgen.1007081 PMC5711033

[24] Lorentzon M, Cummings SR. Osteoporosis: the evolution of a diagnosis. J Intern Med. 2015;277(6):650–61. 25832448 10.1111/joim.12369

[25] Sözen T, Özışık L, Başaran N. An overview and management of osteoporosis. Eur J Rheumatol. 2017;4(1):46–56. 28293453 10.5152/eurjrheum.2016.048 PMC5335887

[26] Verbanck M, Chen CY, Neale B, et al. Detection of widespread horizontal pleiotropy in causal relationships inferred from Mendelian randomization between complex traits and diseases. Nat genet. 2018;50(5):693–8. 29686387 10.1038/s41588-018-0099-7 PMC6083837

[27] Bowden J, Davey Smith G, Burgess S. Mendelian randomization with invalid instruments: effect estimation and bias detection through Egger regression. Int J Epidemiol. 2015;44(2):512–25. 26050253 10.1093/ije/dyv080 PMC4469799

[28] Wang W, Tan JS, Hua L, et al. Genetically predicted obesity causally increased the risk of hypertension disorders in pregnancy. Front Cardiovasc Med. 2022;9:888982. 35694671 10.3389/fcvm.2022.888982 PMC9175023

[29] Burgess S, Thompson SG. Interpreting findings from Mendelian randomization using the MR-Egger method. Eur J Epidemiol. 2017;32(5):377–89. 28527048 10.1007/s10654-017-0255-x PMC5506233

[30] Tan JS, Liu N, Guo TT, et al. Genetic predispositions between COVID-19 and three cardio-cerebrovascular diseases. Front Genet. 2022;13:743905. 35368685 10.3389/fgene.2022.743905 PMC8966609

[31] Burgess S, Thompson SG. Mendelian Randomization: Methods for Causal Inference Using Genetic Variants. 2nd ed. Chapman and Hall/CRC; 2021. 10.1201/9780429324352.

[32] Bowden J, Del Greco MF, Minelli C, et al. A framework for the investigation of pleiotropy in two-sample summary data Mendelian randomization. Stat Med. 2017;36(11):1783–802. 28114746 10.1002/sim.7221 PMC5434863

[33] Ma C, Su H, Li H. Global research trends on prostate diseases and erectile dysfunction: a bibliometric and visualized study. Front Oncol. 2020;10:627891. 33643922 10.3389/fonc.2020.627891 PMC7908828

[34] Chen C. Searching for intellectual turning points: progressive knowledge domain visualization. Proc Natl Acad Sci U S A. 2004;101 Suppl 1(Suppl 1):5303–10. 14724295 10.1073/pnas.0307513100 PMC387312

[35] Arruda H, Silva ER, Lessa M, et al. VOSviewer and Bibliometrix. J Med Libr Assoc. 2022;110(3):392–5. 36589296 10.5195/jmla.2022.1434 PMC9782747

[36] Shrine N, Guyatt AL, Erzurumluoglu AM, et al. New genetic signals for lung function highlight pathways and chronic obstructive pulmonary disease associations across multiple ancestries. Nat Genet. 2019;51(3):481–93. 30804560 10.1038/s41588-018-0321-7 PMC6397078

[37] Malaise O, André C, Van Durme C, et al. Lung function impairment, associating hyperinflation with impaired diffusion capacity and transfer coefficient, is a risk factor for hip osteoporosis in patients with chronic obstructive pulmonary disease. J Clin Med. 2023;12(6):2383. 36983383 10.3390/jcm12062383 PMC10059846

[38] Graat-Verboom L, Smeenk FW, Van Den Borne BE, et al. Risk factors for osteoporosis in Caucasian patients with moderate chronic obstructive pulmonary disease: a case control study. Bone. 2012;50(6):1234–9. 22426499 10.1016/j.bone.2012.02.638

[39] Díez-Manglano J, Berges Vidal M, MartínezBarredo L, et al. Chronic obstructive pulmonary disease and incidence of hip fracture: a nested case-control study in the EpiChron Cohort. Int J Chron Obstruct Pulmon Dis. 2020;15:2799–806. 33177817 10.2147/COPD.S270713 PMC7652231

[40] Romme EA, Murchison JT, Edwards LD, et al. CT-measured bone attenuation in patients with chronic obstructive pulmonary disease: relation to clinical features and outcomes. J Bone Miner Res. 2013;28(6):1369–77. 23361992 10.1002/jbmr.1873

[41] Bon J, Fuhrman CR, Weissfeld JL, et al. Radiographic emphysema predicts low bone mineral density in a tobacco-exposed cohort. Am J Respir Crit Care Med. 2011;183(7):885–90. 20935108 10.1164/rccm.201004-0666OC PMC3086755

[42] Nie H, Wang F, Zeng X, et al. Analysis of communal molecular mechanism between chronic obstructive pulmonary disease and osteoporosis. Int J Chron Obstruct Pulmon Dis. 2023;18:259–71. 36937804 10.2147/COPD.S395492 PMC10017835

