# Supplementary material for: Association between chronic obstructive pulmonary disease and osteoporosis: Mendelian randomization combined with bibliometric analysis
Source: Hereditas. 2025 Feb 1;162:14. doi: 10.1186/s41065-025-00373-z (PMC11787750; doi:10.1186/s41065-025-00373-z)
Supplement: Supplementary file 2 — Supplementary Material 2. [file 41065_2025_373_MOESM2_ESM.zip › Figures/COPD-TB-BMD(Fig12).pdf]

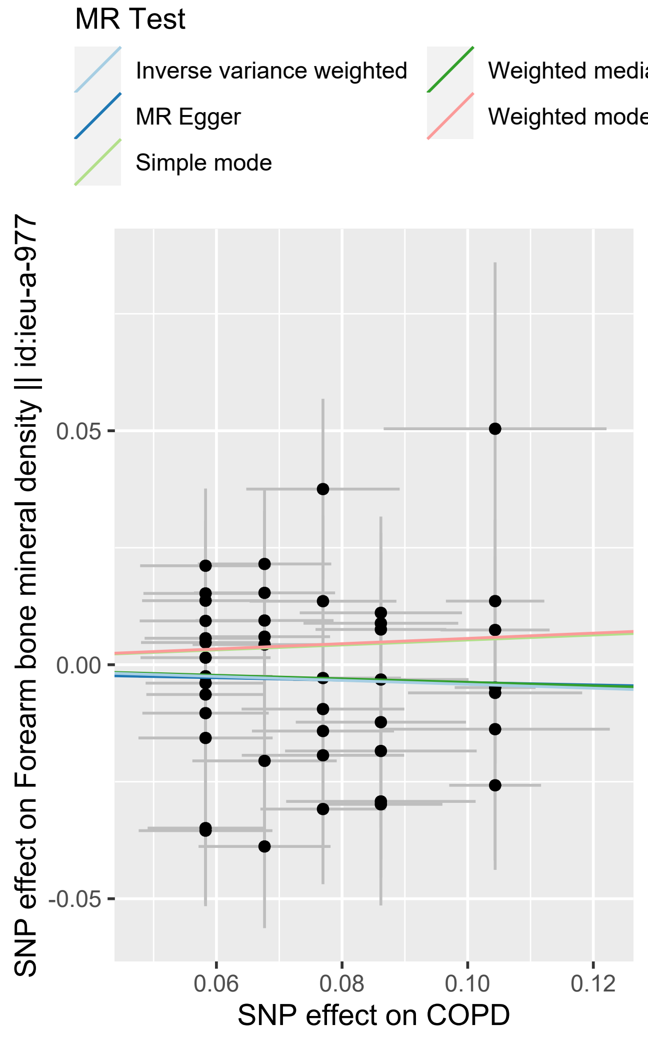
MR Test
Inverse variance weighted
MR Egger
Simple mode
Weighted media
Weighted mode
SNP effect on Forearm bone mineral density || id:ieu-a-977
0.05
0.00
-0.05
0.06
0.08
0.10
0.12
SNP effect on COPD
