# Supplementary figures and images for: Association between chronic obstructive pulmonary disease and osteoporosis: Mendelian randomization combined with bibliometric analysis
Source: Hereditas. 2025 Feb 1;162:14. doi: 10.1186/s41065-025-00373-z (PMC11787750; doi:10.1186/s41065-025-00373-z)

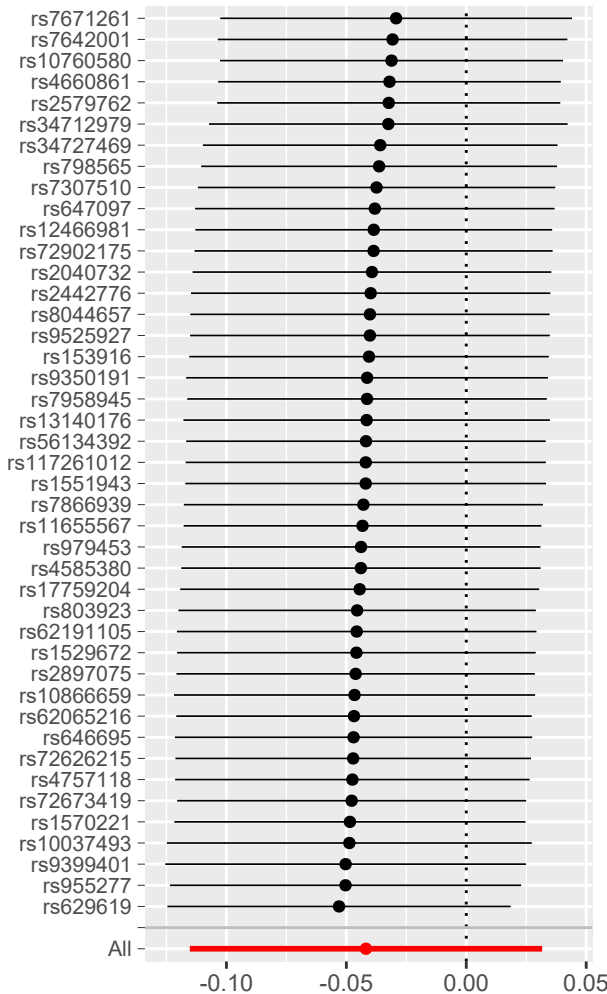

MR leave-one-out sensitivity analysis for  
'COPD' on 'Forearm bone mineral density || id:ieu-a-9

Supplement: Supplementary file 2 — Supplementary Material 2. [file 41065_2025_373_MOESM2_ESM.zip › Figures/COPD-FA-BMD(Fig1).pdf]

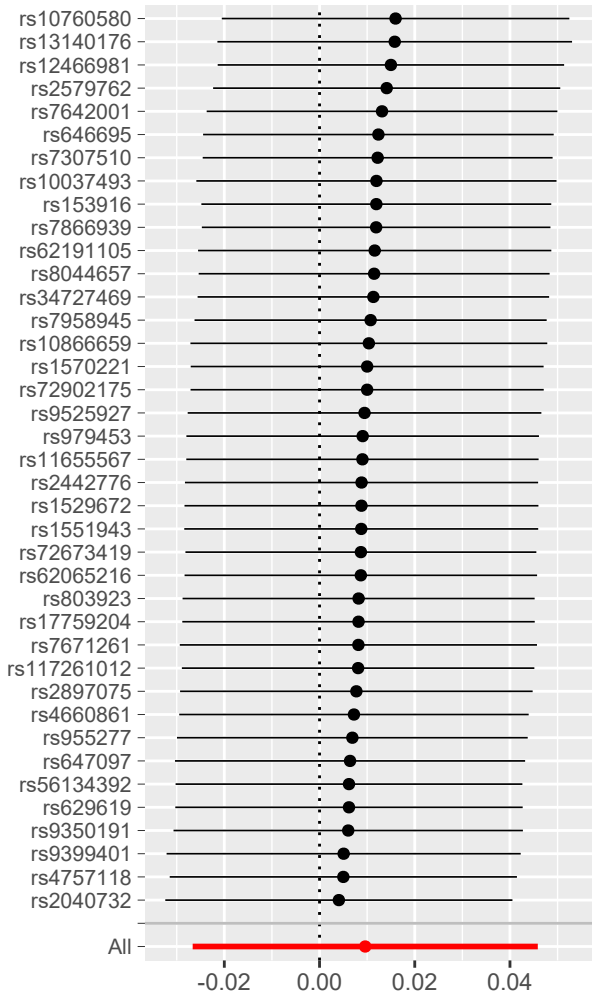

MR leave-one-out sensitivity analysis for  
'COPD' on 'Femoral neck bone mineral density || id:ieu-a'

Supplement: Supplementary file 2 — Supplementary Material 2. [file 41065_2025_373_MOESM2_ESM.zip › Figures/COPD-FN-BMD(Fig2).pdf]

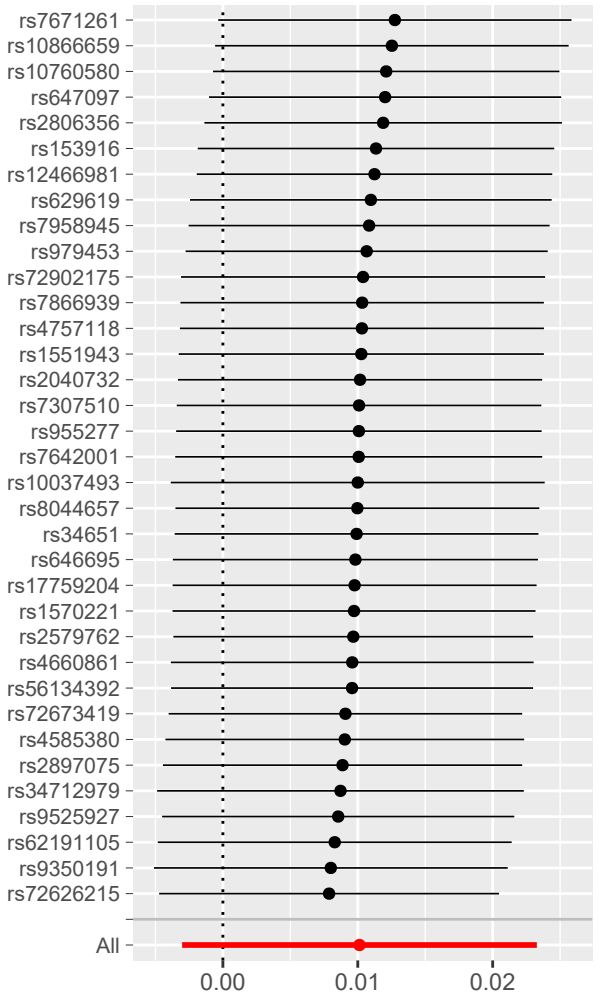

MR leave-one-out sensitivity analysis for 'COPD' on 'Heel bone mineral density || id:ebi-a-GCST00

Supplement: Supplementary file 2 — Supplementary Material 2. [file 41065_2025_373_MOESM2_ESM.zip › Figures/COPD-HE-BMD(Fig3).pdf]

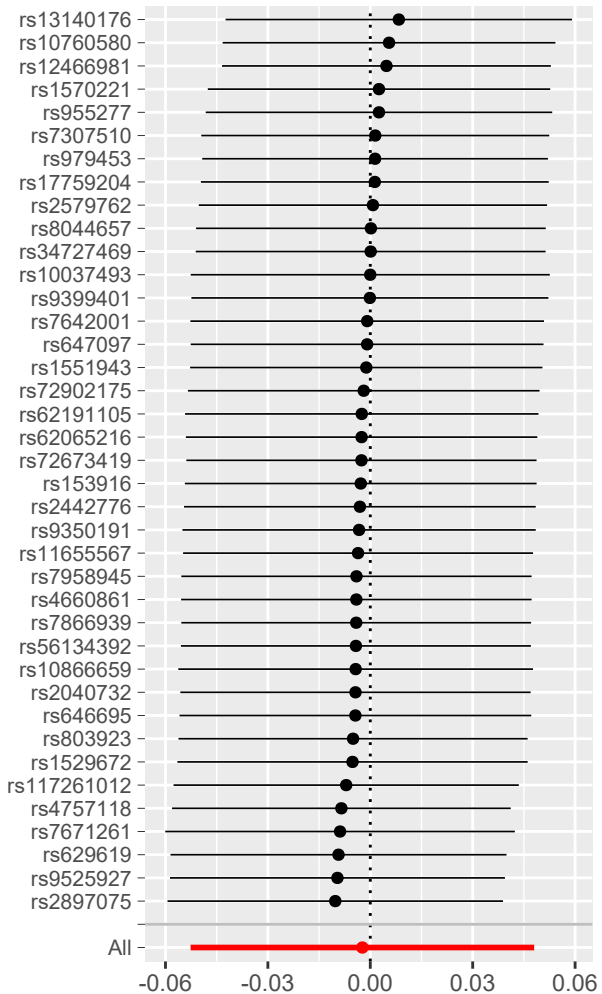

MR leave-one-out sensitivity analysis for  
'COPD' on 'Lumbar spine bone mineral density || id:ieu-a'

Supplement: Supplementary file 2 — Supplementary Material 2. [file 41065_2025_373_MOESM2_ESM.zip › Figures/COPD-LS-BMD(Fig4).pdf]

## MR Method

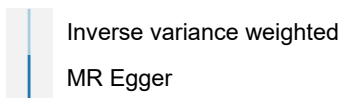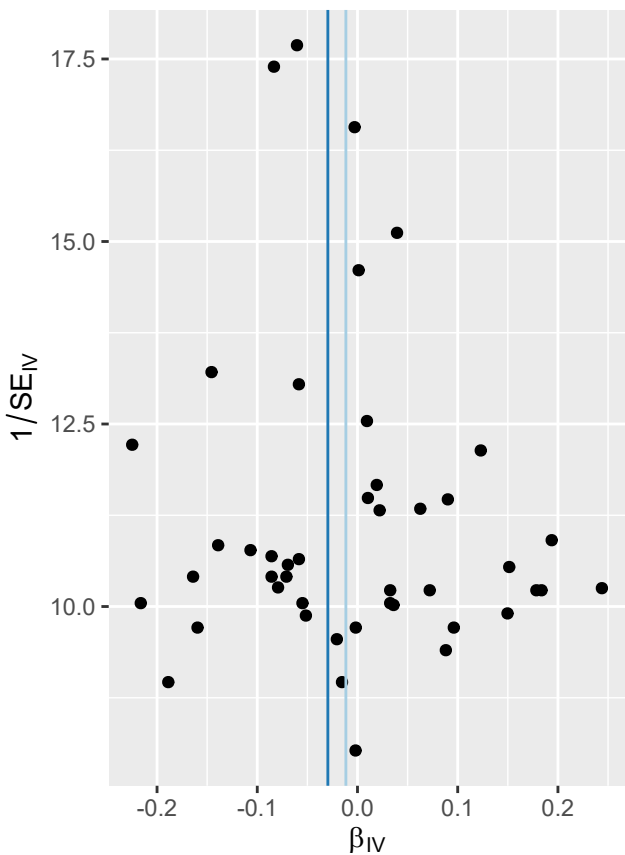

Supplement: Supplementary file 2 — Supplementary Material 2. [file 41065_2025_373_MOESM2_ESM.zip › Figures/COPD-TB-BMD (Fig10).pdf]

# MR Method

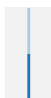

Inverse variance weighted

MR Egger

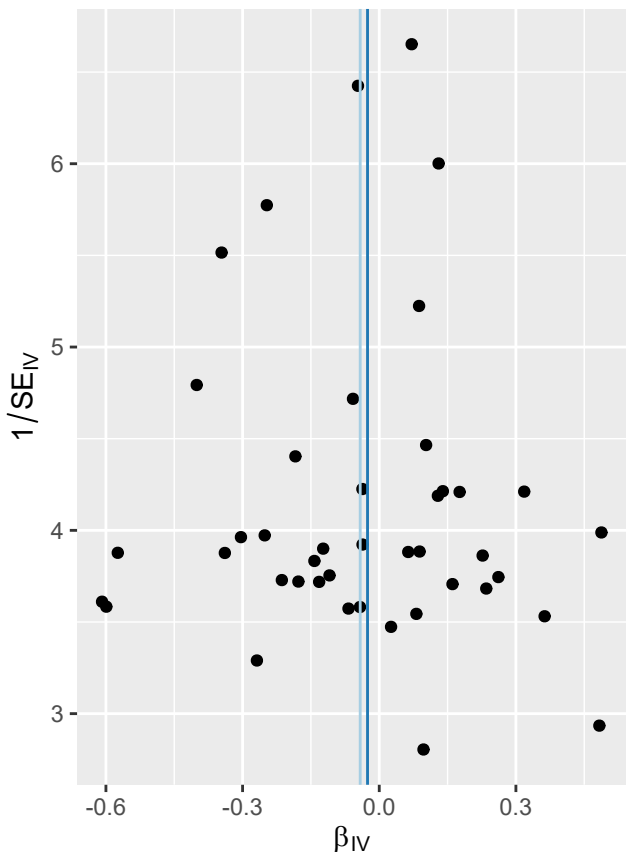

Supplement: Supplementary file 2 — Supplementary Material 2. [file 41065_2025_373_MOESM2_ESM.zip › Figures/COPD-TB-BMD (Fig6).pdf]

## MR Method

Inverse variance weighted

MR Egger

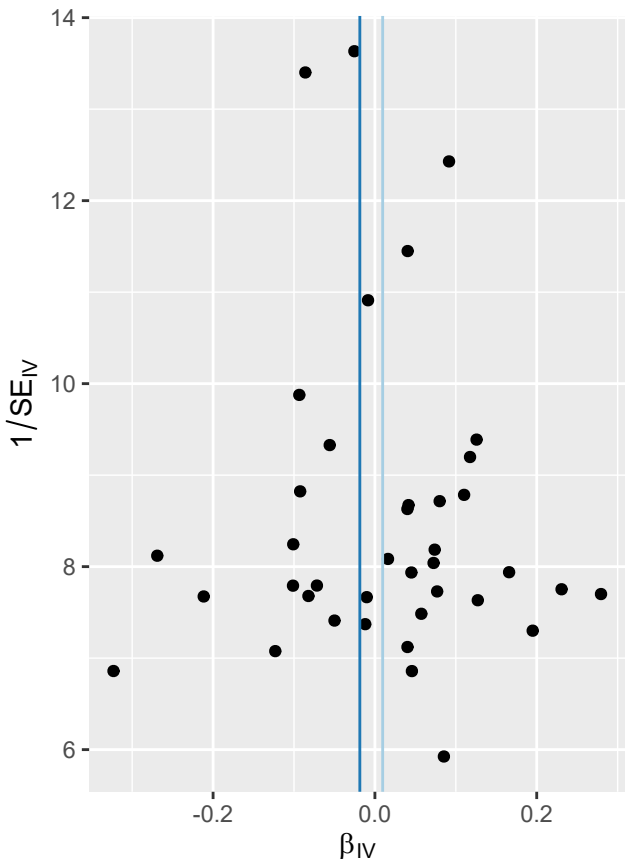

Supplement: Supplementary file 2 — Supplementary Material 2. [file 41065_2025_373_MOESM2_ESM.zip › Figures/COPD-TB-BMD (Fig7).pdf]

## MR Method

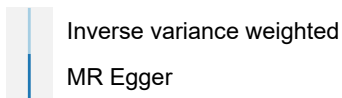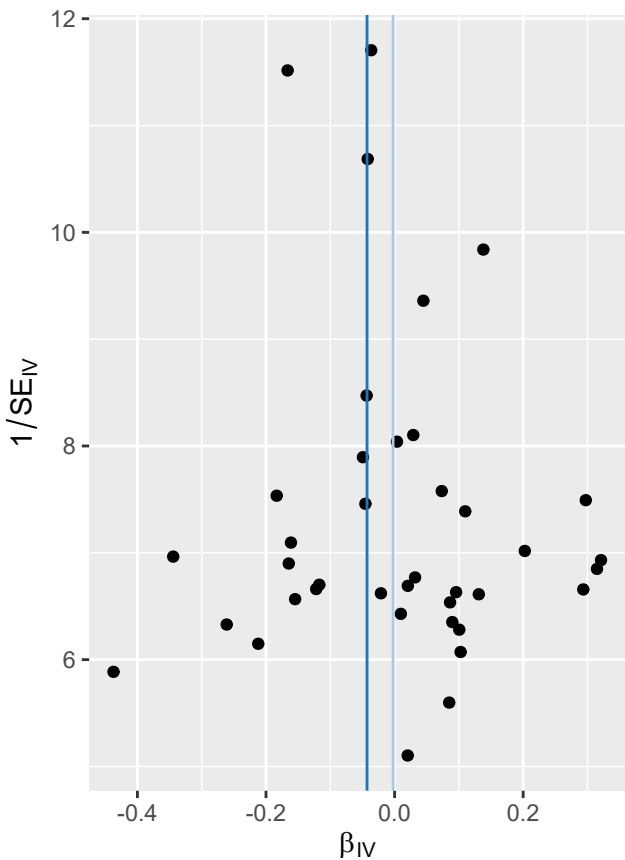

Supplement: Supplementary file 2 — Supplementary Material 2. [file 41065_2025_373_MOESM2_ESM.zip › Figures/COPD-TB-BMD (Fig8).pdf]

## MR Method

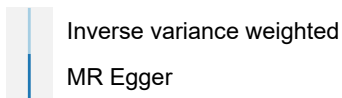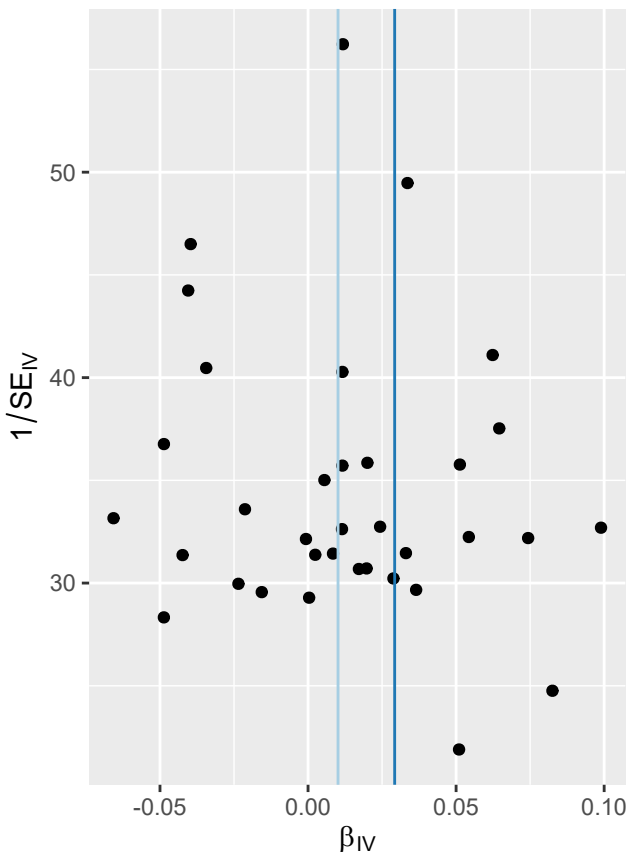

Supplement: Supplementary file 2 — Supplementary Material 2. [file 41065_2025_373_MOESM2_ESM.zip › Figures/COPD-TB-BMD (Fig9).pdf]

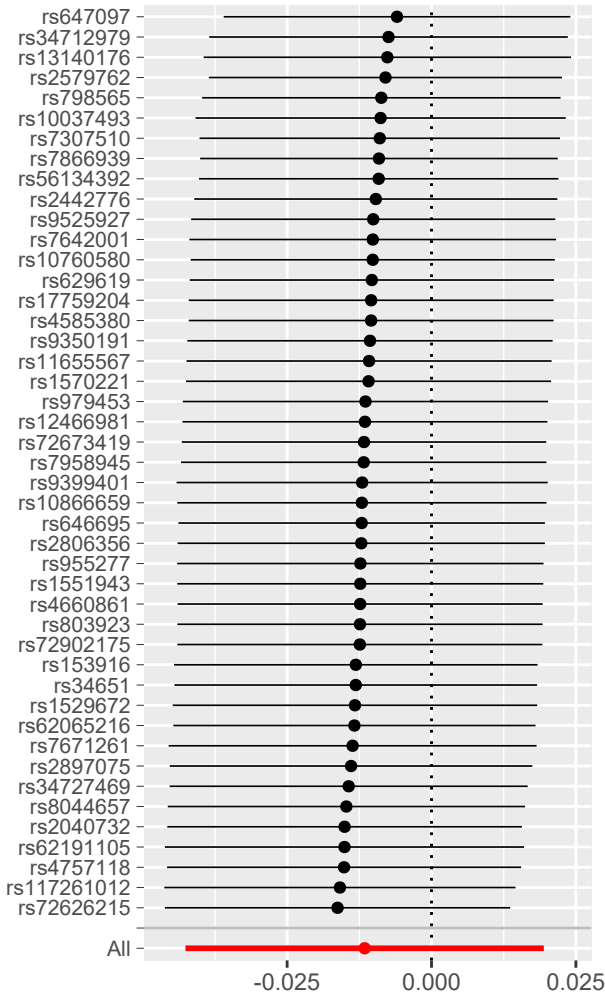

Supplement: Supplementary file 2 — Supplementary Material 2. [file 41065_2025_373_MOESM2_ESM.zip › Figures/COPD-TB-BMD(Fig5).pdf]
